# “Path of the Heart” (The BPROAD Study) Addresses Optimal Systolic Blood Pressure for Patients With Diabetes

**DOI:** 10.1111/1753-0407.70053

**Published:** 2025-01-23

**Authors:** Ning Guang

**Affiliations:** ^1^ Department of Endocrine and Metabolic Diseases, Shanghai Institute of Endocrine and Metabolic Diseases, Ruijin Hospital Shanghai Jiao Tong University School of Medicine Shanghai China; ^2^ National Clinical Research Center for Metabolic Diseases (Shanghai), Key Laboratory for Endocrine and Metabolic Diseases of the National Health Commission of the PR China, National Research Center for Translational Medicine, Shanghai Key Laboratory for Endocrine Tumor, State Key Laboratory of Medical Genomics, Ruijin Hospital Shanghai Jiao Tong University School of Medicine Shanghai China

Awaiting my presentation at the centennial gathering of the American Heart Association (AHA) in Chicago, I opened my diary once again, particularly the one dated February 23, 2019, which reads, “After two years of meticulous preparation, the Path of the Heart research initiative has finally commenced.” The Path of the Heart refers to the BPROAD study, which has garnered significant acclaim due to its presentation at the Late‐Breaking Science session of the AHA and concurrent publication in the prestigious *New England Journal of Medicine* [1]. Yet, few are acquainted with the trepidation that marked the inception of this endeavor 5 years prior, the indecision that lingered during the 2‐year preparation phase, or the challenges posed by the COVID‐19 pandemic throughout the study's execution. I extend my profound admiration and gratitude to the team led by Prof. Wang Weiqing, with Bi Yufang, Xu Yu, and Li Mian at the helm of the core research group, for their indomitable spirit and the resounding success of the study.

Hypertension affects 23.2% of the adult Chinese population, with a staggering half of diabetes patients also suffering from hypertension. Hypertension has emerged as the preeminent cause of mortality and disability among diabetes. Consequently, blood pressure management has become equally as imperative as glycemic control in the therapeutic strategies for diabetes in China. However, the optimal target for blood pressure reduction remains elusive. While the SPRINT study demonstrated a significant reduction in cardiovascular events with systolic blood pressure below 120 mmHg in hypertensive patients without diabetes [2, 3], the ACCORD study failed to observe similar benefits in diabetes patients. Besides, the ACCORD study was a 2 × 2 factorial‐design study examining both blood pressure and glucose control [4]. Therefore, the target for blood pressure reduction in diabetes patients has become an unresolved issue, casting a shadow of confusion over clinical practice.

In light of this, the team led by Wang Weiqing and Bi Yufang from Ruijin Hospital has spearheaded the BPROAD study [5, 6]. This nationwide, multicenter, open‐label, parallel‐group, randomized controlled clinical trial made its debut as the opening presentation at the 2024 AHA Scientific Session, marking a historic milestone for Chinese researchers in the field of cardiovascular and metabolic clinical research.

The BPROAD study has established that intensive blood pressure management targeting a systolic blood pressure below 120 mmHg, as opposed to conventional management aiming for below 140 mmHg, results in a 21% reduction in the primary composite endpoint of major cardiovascular events, including non‐fatal stroke, non‐fatal myocardial infarction, heart failure requiring treatment or hospitalization, and cardiovascular death, in type 2 diabetes patients with elevated systolic blood pressure and increased cardiovascular risk.

This groundbreaking research has the potential to revolutionize treatment protocols. A rough estimate suggests that with ~120 million diabetes patients in China, 70 million of whom have concomitant hypertension, reducing systolic blood pressure from 140 to 120 mmHg could avert 300 000 major cardiovascular events annually, underscoring the study's profound implications. However, stricter blood pressure control necessitates an increase in antihypertensive medication prompting the need for research into antihypertensive drug combinations more suited to diabetes patients.

The BPROAD study also revealed an increased likelihood of hypotension as blood pressure approaches normal levels, necessitating further investigation into how to manage blood pressure in diabetes patients without inducing hypotension. Another concern is electrolyte imbalances, particularly potassium. The use of diuretics raises the risk of hypokalemia, while ACE inhibitors may lead to hyperkalemia. Maintaining electrolyte balance, with a focus on normal potassium levels, is critical in the management of blood pressure in diabetes patients. Urinary albumin excretion, typically measured by the urinary albumin excretion rate or the albumin‐to‐creatinine ratio, is often overlooked by both patients and physicians. This is another critical parameter to consider in the use of antihypertensive drugs for diabetes patients. Therefore, it is imperative to explore blood pressure reduction strategies that are more suitable for diabetes patients, and the development of more comprehensive blood pressure reduction protocols is a research topic that warrants further exploration. Additionally, the BPROAD study noted but did not emphasize the importance of salt reduction. Given the high salt and oil content in Chinese diets, particularly in northern regions, finding dietary solutions that cater to local tastes while promoting health is another research area that requires our attention.

The publication of the BPROAD study signifies both the culmination and the genesis of further inquiry. As researchers, we stand at the intersection of resolution and revelation, for with each question answered, a multitude of new questions emerge. The pursuit of science is boundless, yet to tread the path of scientific truth is both our fortune and our duty. I extend my gratitude to all participants of the BPROAD study and to all those who have supported and shown interest in this study.

## Conflicts of Interest

The author declares no conflicts of interest.
